# Whole Genome Sequencing instead of Whole Exome Sequencing is required to identify the Genetic Causes of Polycystic Ovary Syndrome in Pakistani families

**DOI:** 10.12669/pjms.343.14644

**Published:** 2018

**Authors:** Muhammad Jaseem Khan, Rubina Nazli, Jawad Ahmed, Sulman Basit

**Affiliations:** 1 Muhammad Jaseem Khan, M.Phil. Institute of Basic Medical Sciences, Khyber Medical University, Peshawar, Pakistan.; 2 Dr. Rubina Nazli, MBBS, PhD. Institute of Basic Medical Sciences, Khyber Medical University, Peshawar, Pakistan; 3 Dr. Jawad Ahmed, MBBS, PhD. Institute of Basic Medical Sciences, Khyber Medical University, Peshawar, Pakistan; 4 Dr. Sulman Basit, PhD. Center for Genetics and Inherited Diseases, Taibah University, Almadina Almunawara, Saudi Arabia

**Keywords:** Polycystic ovary syndrome, SNP microarray, Whole exome sequencing

## Abstract

**Background & Objective:**

Polycystic Ovary Syndrome (PCOS) is the major cause of infertility in females. PCOS is a complex and multifactorial disease, genetic and environmental factors being important predisposing factors. Diagnosis of PCOS is difficult due to the complexity of this disease; hence, better diagnostic tests are required to improve its management. Aim of the study was to elucidate the genetic causes of PCOS in three Pakistani families.

**Methods:**

Three Pakistani families segregating PCOS in an apparently autosomal recessive mode were recruited. Whole genome Single Nucleotide Polymorphism (SNP) genotyping and Whole Exome Sequencing (WES) were carried out to identify the candidate genes.

**Results:**

SNP genotypes data analyses identified multiple regions of homozygosity on different chromosomes. WES was performed in affected members of the family. Screening for pathogenic mutations in homozygous regions failed to detect any mutation/variant of interest.

**Conclusion:**

PCOS is multifactorial and complex disease so variants in the coding as well as in non-coding regions may be the genetic causes of the disease. To elucidate the genetic cause(s) of the PCOS, Whole Genome Sequencing (WGS) is recommended to cover both coding and non-coding regions of the genome.

## INTRODUCTION

Polycystic Ovary Syndrome (PCOS) is a major cause of infertility affecting 6-10% of the female population.^1^ Phenotypes are variable, characterized by polycystic ovaries, hyperandrogenism, obesity, ovulatory dysfunction, fecund ability and early pregnancy loss. Due to PCOS, infertility in female is often associated with neurological disorders, cardiac diseases, tumors of breast and endometrium.^2^,^3^ PCOS is a known cause of an ovulatory infertility in women.^4^ Etiology of the PCOS still remains inconclusive, however; it is evident that both genetic and environmental factors are involved.^5^,^6^

PCOs can lead to irregular menstrual cycle and infertility. It leads to anovulation in about 90% of patients.^7^ In PCOS the oogenesis process is different as compared to normal cycling fertile woman. The gonadotropins cannot affect the activation from primordial to primary follicle hence the adjacent granulosa cells gets androgen from the theca cell layer. In addition, LH stimulation converts androgen to estradiol. Therefore, these two factors, LH and estradiol, are significantly altered in PCOS patients.^8^-^11^

Diagnosis of PCOS is difficult because of the different diagnostic parameters. Some of these parameters may be altered while other remain normal. In 60% cases of PCOS the elevated levels of the androgen is considered to be the standard diagnostic test.^12^ However, inconsistent results make it unreliable test. Similarly, Sex Hormone Binding Globulin (SHBG) protein and testosterone can also help in the diagnosis of PCOS but may yield unreliable results^13^. Additionally, ultrasonography also helps in the diagnosis of PCOS. Multi-follicular ovarian morphology on ultrasonography is the characteristic feature of PCOS. Since there is difficulty in the diagnosis of PCOS, a better evaluation method and diagnostic tests are required.

There are several hypotheses for the genetic predisposition to PCOS. The involvement of genetic factors has been elucidated in several studies. Many case-control studies have showed the association of certain genetic variants with the disease but the replication studies failed to show consistent results due to lack of sufficient sample sizes.^14^ Several studies have showed an association of variants in the Insulin, Insulin Receptor (INSR) and SHBG genes with PCOS.^15^

Variants associated with PCOS can be delineated by using an approach of Genome-Wide Association Study (GWAS). Few GWAS in PCOS patients have been performed to date.^16^,^17^ The first locus identified through GWAS is on chromosome 2p16.3. This locus contains two candidate genes including testis specific gene *GTF2A1L* and LH receptor encoding gene *LHCGR*. *LHCGR* gene plays a critical role in ovulation and pregnancy maintenance. Another locus was identified through GWAS on chromosome 9q33.3. *DENND1A* gene in this locus was considered as a candidate gene based on its role in regulating the production of endoplasmic reticulum amino-peptidase-1, used for membrane trafficking.^18^

Most recent GWAS and meta-analysis of candidate genes showed significant association of two variants in the *FBN3* gene with PCOS phenotype.^19^ FBN3 gene encodes fibrillin-3 protein. Fibrillin-3 is a structural component of connective tissues.^20^

Difficulties in recruiting larger families from advanced countries and lack of advance genetic screening in Pakistan enabled us to design the present study to find out possible genetic cause(s) of PCOS in Pakistani families using WGS and WES.

## METHODS

This study was approved by the Ethical Review Board, Khyber Medical University Peshawar and is in accordance with Helsinki declaration. All families were recruited according to the androgen excess society guidelines. Proband demonstrate both:

- Hirsutism and/or hyperandrogenemia,
- Oligoanovulation and/or polycystic ovaries. All other etiologies were excluded i.e. due to androgen excess or anovulation from probands.

**Fig.1:**
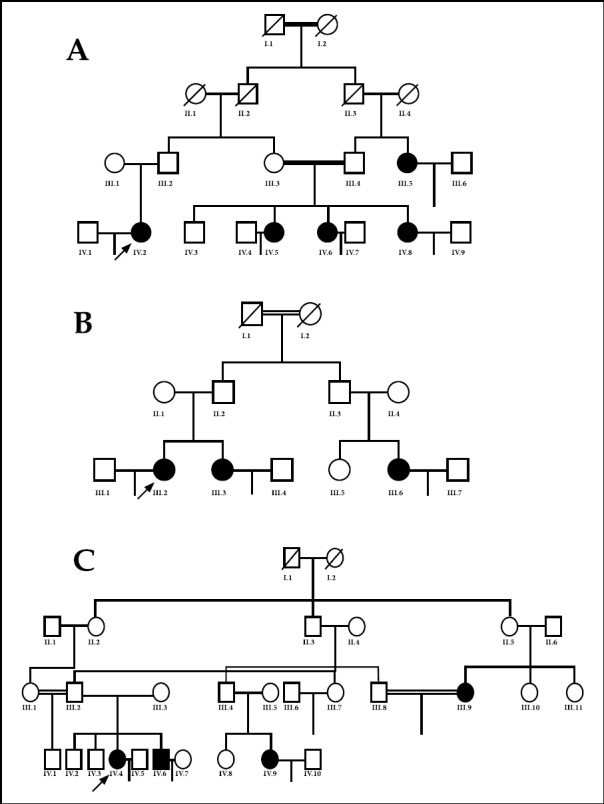
Pedigrees of families A, B and C. The affected individuals are shown with black filled square and circles. The diagonal line crossing indicates the deceased individuals. The double line indicates cousin marriages and the infertility is proven by no offspring with empty line.

Three Pakistani families with likely autosomal recessive PCOS (Figures 1a, 1b and 1c) were enrolled in this study after obtaining informed consent. Detail family history and clinical examination, followed by ultrasonography of the affected participants was carried out. Whole blood was collected for genetic analysis and serum for hormone analysis.

### Whole genome genotyping

Genomic DNA was extracted with standard phenol-chloroform method as described previously.^21^ All the affected individuals, normal sibling and parents in each family were genotyped using Illumina Human Omni 2.5M BeadChip containing 2,500,000 SNPs (Single Nucleotide Polymorphisms). Genomic DNA (200ng) was denatured with 0.1N NaOH and whole genome amplification was carried out with Random Primers Mix (RPM) using Multi Sample Master Mix (MSM). The amplified DNA was enzymatically fragmented using Fragmentation Mix (FMS) followed by precipitation using Precipitation Mix 1 (PM1) and 2-propanol. Hybridization of fragmented DNA to Bead Chip was performed by denaturing the sample and dispensing 35 ul of sample onto the BeadChip section followed by incubation for 18hr at 48°C in the hybridization oven. BeadChips were washed and staining was performed following single base extension. This reaction incorporates labeled nucleotides into the extended primers. Imaging was performed in Illumina iScan scanner using iScan control software. Homozygosity Mapper was used to detect genome wide shared homozygous regions. Illumina Genome Viewer software incorporated in Genome Studio was used to detect copy number variations (CNVs) in the genome.

### Whole Exome Sequencing

Two affected and one normal participant from each family was subjected to exome sequencing. Nextera Rapid Capture Exome kit was used for library preparation and exome enrichment. Cluster generation and DNA sequencing was performed on Illumina NextSeq500 instrument.

Briefly, 50ng of DNA was fragmented using enzymatic method followed by tagmentation. Libraries were purified with magnetic beads and target regions were captured with whole exome oligos followed by PCR amplification of the enriched library. Library was quantified with Qubit fluorimeter and library size distribution was measured with Agilent Bioanalyser. BWA aligner incorporated in BaseSpace was used to align fastq files to the reference genome using the BWA-MEM algorithm. Variants were called using genome analysis tool kit (GATK). Illumina Variant Studio was used for annotation and filtration of the genomic variants. Sanger sequencing was performed for variants of interest to confirm the variants discovered by WES. Primer-3 software (http://frodo.wi.mit.edu/primer3/) was used to design primers for PCR amplification of the variants and their flanking regions. BIOEDIT sequence alignment editor version 6.0.7 (Ibis Biosciences Inc., Carlsbad, CA, USA) was used for sequence alignment.

## RESULTS

### Clinical picture of affected individuals

All the affected members of the recruited families underwent extensive medical investigations. All affected participants had a history of infertility for the last four years after marriage. Their hormone analysis showed altered levels with raised serum testosterone levels (Table-I).

**Table-I:** Details of the Hormone analysis of the affected family members.

Assays	Family A			

	Case ID			
	IV:2	IV:5	IV:6	IV:8
Luteinizing hormone	3.26	5.39	4.69	15.09
Follicle stimulating hormone	0.15	2.18	4.64	6.03
Testosterone	81.04	75.43	44.53	72.72

	**Family B**			

	III:2	III:3	III:6	
Luteinizing hormone	5.95	15.34	9.45	
Follicle stimulating hormone	2.84	5.64	4.02	
Testosterone	108.15	17.09	71.49	

	**Family C**			

	III:9	IV:4	IV:9	
Luteinizing hormone	33.32	47.75	35.26	
Follicle stimulating hormone	53.36	6.87	10.56	
Testosterone	42.43	34.46	39.09	

***Family A:*** Proband (IV-2) has irregular menstrual cycle and oligoovulation. Ultrasonographic examination revealed small size uterus. Right ovary was larger in size with multiple follicles while the left ovary was normal in size but the follicular activity was absent. The endometrium was distorted with focal glandular and stromal breakdown.

All the affected probands (IV-2, IV-5, IV-6, IV-8) have high levels of testosterone (81.04, 75.43, 44.53, 72.72 ng/dl). Hyperandrogenism with oligoovulation confirms the diagnosis of PCOS.

***Family B:*** Ultrasonographic examination of the participants III-2 and III-3 revealed bilateral patent fallopian tubes with the history of irregular menstrual cycle and oligomenorrhea. Proband III-6 had a small size uterus with desquamated endometrium. Hormone analysis revealed high levels of testosterone [(III-2, 108), (III-3, 71.9) and (III-6, 71.49)] and fatty abdomen of all affected probands.

***Family C:*** Ultrasonographic examination of the proband IV-4 in this family revealed patent tubes and anovular ovaries with a history of dysmenorrhea. Proband III-9 had hirsutism and low pitched voice. A high level of testosterone in all the three affected participants i.e [(III-9, 62.43), (IV-4, 74.46) and (IV-9, 59.9)] confirmed the diagnosis of PCOS.

### SNP microarray failed to identify conclusive homozygous region

Whole genome homozygosity mapping was carried out using Illumina 2.5M array. DNA of nine affected individuals; family A four (IV-2, IV-5, IV-6, IV-8), family B three (II-2, III-3, III-6) and family C two (IV-4, IV-9) affected individuals, parents and normal individuals was used for detection of Loss of Heterozygosity (LOH) regions. Genotype data analyses using Homozygosity Mapper^22^ identified multiple homozygous regions in affected individuals in all three families.

### Whole exome failed to identify mutation

Two affected and two normal participants were selected for WES. The sequencing identified millions of unique variants; the variants included single nucleotide variation and small insertion/deletion. Those variants which were functional and their frequency was <0.01% were selected. The genes were further screened on the basis of pedigree information and involvement in infertility. Thereby, we identified homozygous mutations in family A (IV-2, IV-5), family B (III-3, III-6) and family C (III-9, IV-4) in genes mentioned in Table-II. However, segregation analysis using DNA samples from unaffected members of each family failed to show the segregation of variants with disease phenotype. Therefore, variants identified in this study are not the underlying cause of PCOS in these families.

**Table-II:** List of genes identified and extracted from WES data.

Family A			

Gene	Coordinate site	Chromosome	Change
MAML3	140811063	4	TTGCTGCTGCTGC>T/T
PCGF6	105110818	10	C>A/A
TFAP2C	55204652	20	A>T/T

**Family B**			

NBPF16	148756664	1	G>T/T
ACTL9	8808648	19	T>G/G
CACNA1A	13318672	19	CCTGCTG>C/C
DOCK4	111368395	7	C>A/A
CPEB2	15004878	4	A>AGCCGCC/AGCCGCC

**Family C**			

AMDHD2	2570497	16	T>G/G
OR2T35	248801602	1	T>TCA/TCA
LRP1	57569490	12	T>G/G

## DISCUSSION

The present study was designed to find out the familial cause of the PCOS. Hormonal, ultrasonographic and clinical examination confirms the diagnosis of PCOS. Genotype data analyses using HomozygosityMapper failed to reveal homozygous regions. Proceeding with the genetic analysis, WES and filtering for coding variants present in homozygous or compound heterozygous state in all affected members, present in heterozygous state in the unaffected parents and not present in homozygous or compound heterozygous state in the unaffected sibling did not yield any candidate variant.

Evidence of genetic, environmental and hormonal involvement have been reported for PCOS in a number of studies.^23^,^24^ High heritability (*h2* = 0.70) have been proven in Dutch twin study.^6^ Nevertheless, the mode of heritance of PCOS remains unclear, and both dominant and multigenic modes of transmission have been proposed.^6^,^25^ Although genetic involvement has been proven in PCOS and association studies also indicate the involvement of the risk loci^26^ yet no definitive genetic mutations have been reported for PCOS so far. In the case of an autosomal recessive inheritance combined with strong genetic heterogeneity, our study design failed to detect the genetic origin. WES has limitations regarding coverage^27^ and causative variants outside of the targeted exome; indels, copy number variations, inversions, or translocations can remain undetected. Furthermore, despite our efforts to discover somatic mutations in our data, our analysis was limited by both the read depth in our study, where only variants with an allele frequency of >0.10 or higher would be detected, and also by the availability of only DNA from whole blood, in which the mutation may not even be present. As PCOS is a complex disorder, the involvement of the non coding region may be assumed especially in case where WES fails to identify causative mutations.

## CONCLUSIONS

The variants, identified in this study, were subjected to segregation analysis. Segregation analysis excluded involvement of the all exome discovered variants as an underlying cause of PCOS in these families. This highlight the involvement of non-coding region variants as an underlying cause of PCOS. In such case, WGS is recommended to screen promoters, enhancers and intronic part of the human genome.
